# Cracking the Chameleon: Uncommon Presentations and Pitfalls of Oral Squamous Cell Carcinoma

**DOI:** 10.7759/cureus.106012

**Published:** 2026-03-27

**Authors:** Tejasvini Chauhan, Preety Garg, Prachi Saxena, Jyoti Singh

**Affiliations:** 1 Pathology, School of Medical Sciences and Research, Greater Noida, IND; 2 Pathology, ESIC Hospital, Indore, IND

**Keywords:** atypical presentation, cancer prognosis, misdiagnosis, oral squamous cell carcinoma, treatment delays

## Abstract

Oral squamous cell carcinoma (OSCC) is classically regarded as a malignancy of older adults over the age of 50 with well-defined etiological associations, including tobacco use, alcohol consumption, and betel nut chewing. Increasingly, however, OSCC is being identified in younger individuals who lack these traditional risk factors and present with subtle or atypical clinical features, contributing to diagnostic delays and potentially poorer outcomes. This case series reports three young patients with OSCC who exhibited unusual presentations, underscoring the need for heightened clinical suspicion in this population. A 29-year-old male patient presented with a persistent burning sensation on the lateral border of the tongue that was initially misdiagnosed as an aphthous ulcer; failure of conservative management prompted biopsy, which revealed moderately differentiated OSCC. A 35-year-old male patient presented with unexplained tooth mobility initially attributed to periodontal disease; subsequent development of a nonhealing ulcer led to the diagnosis of well-differentiated OSCC. A 21-year-old female patient presented primarily with cervical lymphadenopathy and minimal intraoral findings; fine-needle aspiration cytology confirmed metastatic OSCC originating from the gingivobuccal sulcus. These cases highlight the diagnostic challenges posed by OSCC in young adults and emphasize the importance of including malignancy in the differential diagnosis of persistent or unexplained oral symptoms, regardless of patient age or risk factor profile. Early biopsy and histopathological confirmation remain critical for timely diagnosis and improved prognosis. Recognition of emerging etiological contributors, including human papillomavirus infection, genetic susceptibility, and chronic local irritation, may further aid in understanding the changing epidemiology of OSCC in younger populations.

## Introduction

Globally, cancer of the lip and oral cavity is the 15th most common cause of cancer-related deaths [1]. Globally, an estimated 188,000 people died from lip and oral cavity cancers in 2022, with men making up the majority, around 131,000 deaths, compared to approximately 58,000 among women [2]. Oral squamous cell carcinoma (OSCC) is commonly linked to characteristic clinical and histopathological patterns and is most frequently diagnosed in older individuals with established risk factors like tobacco and alcohol consumption [3]. While OSCC is more commonly seen in older adults with known risk factors, it may also occur in younger individuals without such histories, often presenting with diverse and atypical features that complicate timely diagnosis [4]. This article delves into the diverse and often deceptive clinical behavior of OSCC, shedding light on its atypical forms-such as lesions that mimic benign ulcers, nodular growths, unresolved periodontal conditions, or even isolated cervical lymphadenopathy without an obvious primary lesion. By reviewing existing literature with increasingly reported cases of OSCC in younger individuals without traditional risk factors, often presenting with atypical clinical features that may delay diagnosis, and discussing illustrative case examples, this work aims to enhance clinical vigilance among healthcare professionals, emphasizing the need for early suspicion and thorough evaluation, even when OSCC presents in nontraditional or misleading ways. Additionally, genetic susceptibility and hereditary factors have been proposed as potential contributors in such cases, particularly in the absence of known environmental exposures, warranting further investigation into underlying molecular mechanisms.

## Case presentation

Case 1

**Figure 1:**
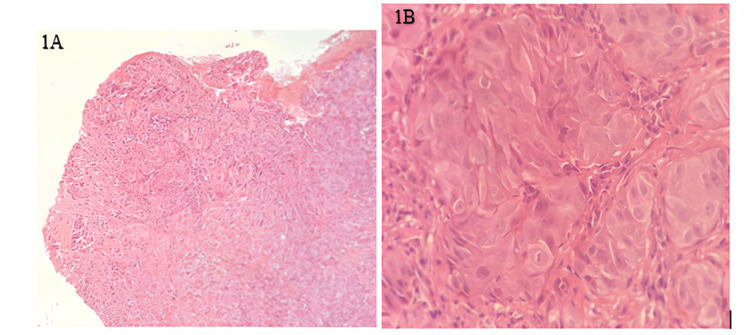
(A) H&E stain 100X; (B) H&E stain 400X, clusters of atypical squamous cells H&E: hematoxylin and eosin

A 29-year-old male patient presented with a persistent burning sensation on the left lateral border of the tongue for three months. On examination, a shallow ulcer measuring approximately 1.2 × 0.8 cm was noted, with an erythematous base, well-demarcated margins, and no induration. The patient reported mild tenderness, but there was no bleeding or exophytic growth. There was no significant family history of malignancy. Based on clinical presentation, differential diagnoses included aphthous ulcer, traumatic ulcer, and oral candidiasis before biopsy was performed. The lesion was initially managed as an aphthous ulcer for two weeks without improvement. Given the persistent nature of the lesion, a biopsy was performed. Histopathological examination revealed sheets and clusters of atypical squamous cells infiltrating the stroma, confirming moderately differentiated squamous cell carcinoma. The patient underwent a partial glossectomy (Figures 1A-1B).

Case 2

**Figure 2:**
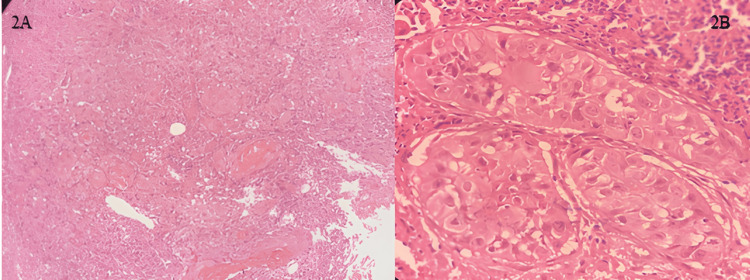
(A) H&E stain 100X, sheets of atypical cells; (B) H&E stain 400X, well-differentiated SCC H&E: hematoxylin and eosin; SCC: squamous cell carcinoma

A 35-year-old male patient presented with a two-month history of unexplained tooth mobility in the lower left jaw. Clinical examination revealed a nonhealing ulcer in the lower gingivobuccal sulcus measuring approximately 1.5 × 1.0 cm. The lesion had slightly indurated margins, a fibrinous exudate covering, and minimal keratotic changes. There was no significant family history of malignancy, associated pain, or obvious mass initially, leading to a differential diagnosis that included periodontal disease, periapical pathology, and localized infection, and was initially treated as periodontal disease secondary to plaque accumulation. A biopsy of the lesion demonstrated dysplastic squamous epithelium with keratinization, and subsequent wide local excision confirmed well-differentiated squamous cell carcinoma (Figures 2A-2B).

Case 3

**Figure 3:**
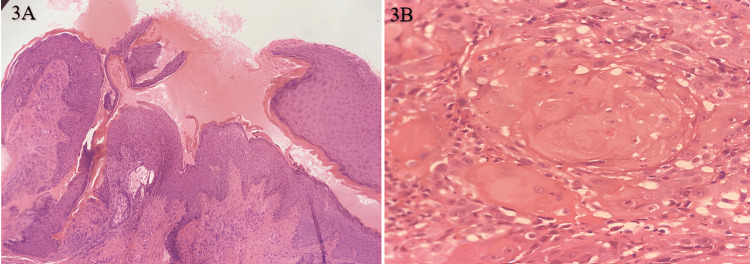
(A) An H&E stain 100 X, low power showing tumor cells infiltrating the dermis; (B) H&E stain 400X, keratin pearl formation H&E: hematoxylin and eosin

A 21-year-old female patient presented with left cervical swelling, gradually enlarging over several weeks. Intraoral examination revealed multiple diminutive reddish papules (~0.2-0.3 cm) in the left gingivobuccal sulcus. These lesions were smooth, nontender, and had intact overlying mucosa. There was no significant family history of malignancy. The cervical lymph node was firm, measuring 1.6 × 1.2 × 0.1 cm, and this cervical lymphadenopathy was initially thought to be due to infectious etiology, granulomatous disease, or reactive in nature and was treated for reactive lymphadenopathy initially. Fine-needle aspiration cytology from the cervical node revealed metastatic squamous cells with a high nuclear-to-cytoplasmic ratio and irregular nuclear contours. An incisional biopsy of the gingival lesions demonstrated keratin pearl formation with marked cellular atypia, confirming metastatic squamous cell carcinoma with the primary lesion in the gingivobuccal sulcus (Figures 3A-3B). The patient underwent surgical excision with nodal dissection.

Clinicopathological characteristics of OSCC cases in young adults have been shown in Table 1. 

**Table 1:** Clinicopathological characteristics of OSCC cases in young adults OSCC: oral squamous cell carcinoma

Parameter	Case 1	Case 2	Case 3
Age/sex	29/M	35/M	21/F
Clinical/gross presentation	Persistent burning sensation on the lateral tongue, erythematous base, no induration or bleeding. Well-demarcated, shallow ulcer measuring 1.2 × 0.8 cm	Unexplained tooth mobility. Nonhealing ulcer measuring 1.5 × 1.0 cm in the lower gingivobuccal sulcus, with slightly indurated margins, fibrinous exudate, and minimal keratotic changes	Nontender, intact mucosa; firm left cervical lymphadenopathy. Multiple smooth, tiny reddish papules measuring 0.2-0.3 cm in the left gingivobuccal sulcus
Provisional diagnosis	Aphthous ulcer	Periodontal disease	Reactive lymphadenopathy
Final diagnosis	Moderately differentiated squamous cell carcinoma	Well-differentiated squamous cell carcinoma	Metastatic squamous cell carcinoma (primary in the gingivobuccal sulcus)
Risk factors	None	None	None
Histopathological findings	Sheets and clusters of atypical squamous cells infiltrating the stroma	Dysplastic squamous epithelium with keratinization	Keratin pearl formation with marked cellular atypia
Treatment	Partial glossectomy	Wide local excision	Surgical excision with nodal dissection

## Discussion

OSCC typically affects individuals over the age of 50 and typically presents with well-characterized lesions such as persistent ulcers, indurated masses, or exophytic growths, often associated with established risk factors like tobacco, alcohol, and betel nut use. However, the cases presented in this report demonstrate that OSCC can also occur in young adults, often without classical risk factors, and present with atypical clinical presentations that may mimic benign or inflammatory oral conditions. These unusual manifestations can delay diagnosis and treatment, ultimately affecting prognosis [5].

In case 1, the lesion was misdiagnosed as an aphthous ulcer and treated conservatively. The failure to respond to therapy prompted further evaluation, and histopathological analysis confirmed moderately differentiated squamous cell carcinoma. This case showed how OSCC in younger patients can be initially overlooked due to subtle symptoms and benign clinical appearance.

Case 2 presented with tooth mobility, a symptom frequently attributed to periodontal disease. The diagnosis of dental plaque masked the underlying pathology until a nonhealing ulcer was discovered on subsequent examination. Histopathology revealed a well-differentiated OSCC. This case highlights that unexplained tooth mobility, particularly in younger patients without significant periodontal risk factors, should not be presumed to result solely from periodontal disease. Instead, it may represent an early clinical indicator of underlying malignancy, including OSCC. The absence of classic inflammatory signs or failure to respond to conventional periodontal therapy should prompt further evaluation through imaging and biopsy, thereby preventing delays in diagnosis [6].

In case 3, cervical lymphadenopathy was the initial complaint. The oral findings, multiple, tiny, nontender gingival papules, were subtle and nonspecific. Fine-needle aspiration cytology from the cervical node revealed metastatic squamous cells, and subsequent incisional biopsy confirmed the primary lesion in the gingivobuccal sulcus. This initial lymph node-dominant presentation represents a biologically important and clinically challenging pattern of OSCC. It may indicate early metastatic potential or an aggressive tumor phenotype, even when the primary lesion is small or clinically inconspicuous. Such presentations carry a significant risk of delayed diagnosis, as the absence of overt intraoral pathology may lead clinicians to initially consider benign or reactive causes of lymphadenopathy. Therefore, persistent or unexplained cervical lymph node enlargement should prompt comprehensive evaluation, including meticulous intraoral examination, appropriate imaging, and timely biopsy to identify occult primary lesions [7].

These three cases collectively showed the diagnostic challenges associated with OSCC in young adults. Atypical presentations such as persistent burning sensation, unexplained tooth mobility, nonhealing gingival lesions, and isolated cervical lymphadenopathy can easily be mistaken for benign or inflammatory conditions, leading to delays in definitive diagnosis. Furthermore, the absence of traditional risk factors in these patients reinforces the growing need to recognize emerging nonconventional etiological contributors, such as human papillomavirus (HPV), genetic predisposition, hereditary predisposition, and possible familial cancer syndromes and chronic mechanical irritation, such as repeated trauma from malocclusion, dental prostheses, sharp teeth, or habitual habits. Although no significant family history was reported in these patients, the absence of detailed genetic testing limits the ability to fully exclude hereditary predisposition. In particular, hereditary factors and underlying genetic alterations may play a crucial role in early-onset cases, especially in patients without identifiable environmental risk factors, warranting further investigation into familial history and molecular pathways involved in tumorigenesis [8].

A notable limitation of the present case series is the absence of molecular or viral testing, including HPV and p16 status, which could have provided further insight into the etiopathogenesis of OSCC in these young patients. Given the emerging role of HPV infection in head and neck carcinogenesis, particularly among individuals without traditional risk factors, incorporation of such analyses in future studies may enhance understanding of tumor biology and guide targeted management strategies. 

Delayed diagnosis of OSCC can significantly worsen patient outcomes, as lesions may progress to advanced stages with deeper tissue invasion and regional lymph node metastasis. This often necessitates more extensive surgical intervention and adjuvant therapy, leading to increased morbidity, functional impairment, and reduced overall survival. Early detection, therefore, plays a pivotal role in improving prognosis and quality of life. Early recognition of OSCC, regardless of patient age or risk profile, is crucial for improving outcomes. Clinicians and dental practitioners must maintain a high index of suspicion when encountering nonresolving oral lesions, particularly those lasting more than two weeks. Prompt biopsy and histopathological confirmation remain the gold standard for early detection [9]. 

Although these cases underscore the occurrence of OSCC in young adults without conventional risk factors, the small series does not allow definitive conclusions regarding temporal trends. Larger studies are needed to assess whether there is a true increase in incidence among younger populations.

## Conclusions

These cases emphasize the dynamic clinical landscape of OSCC, wherein young patients, frequently devoid of conventional risk factors, exhibit subtle, misleading, or atypical symptoms. Misdiagnosis or delayed recognition can profoundly affect treatment outcomes. Symptoms such as burning sensations, unexplained tooth mobility, nonhealing gingival lesions, or isolated cervical lymphadenopathy should not be trivialized in younger individuals, particularly when these manifestations persist despite routine management. A high degree of clinical suspicion, coupled with timely biopsy and histopathological evaluation, remains essential for early diagnosis. These findings suggest the need for increased awareness among dental and medical practitioners regarding the diverse presentations of OSCC, regardless of patient age or risk profile. Early detection is critical, as it directly influences prognosis, therapeutic decisions, and quality of life.
